# ADataViewer: exploring semantically harmonized Alzheimer’s disease cohort datasets

**DOI:** 10.1186/s13195-022-01009-4

**Published:** 2022-05-21

**Authors:** Yasamin Salimi, Daniel Domingo-Fernández, Carlos Bobis-Álvarez, Martin Hofmann-Apitius, Colin Birkenbihl, for the Alzheimer’s Disease Neuroimaging Initiative, the Japanese Alzheimer’s Disease Neuroimaging Initiative, for the Aging Brain: Vasculature, Ischemia, and Behavior Study, the Alzheimer’s Disease Repository Without Borders Investigators, for the European Prevention of Alzheimer’s Disease (EPAD) Consortium

**Affiliations:** 1 Department of Bioinformatics, Fraunhofer Institute for Algorithms and Scientific Computing (SCAI), 53754 Sankt Augustin, Germany; 2 Bonn-Aachen International Center for IT, Rheinische Friedrich-Wilhelms-Universität Bonn, 53115 Bonn, Germany; 3 University Hospital Ntra. Sra. de Candelaria, Santa Cruz de Tenerife, 38010 Spain

**Keywords:** Alzheimer’s disease, Dementia, Data harmonization, Semantic mapping, MRI, Variable catalog, Interoperability, Data curation, Cohort study

## Abstract

**Background:**

Currently, Alzheimer’s disease (AD) cohort datasets are difficult to find and lack across-cohort interoperability, and the actual content of publicly available datasets often only becomes clear to third-party researchers once data access has been granted. These aspects severely hinder the advancement of AD research through emerging data-driven approaches such as machine learning and artificial intelligence and bias current data-driven findings towards the few commonly used, well-explored AD cohorts. To achieve robust and generalizable results, validation across multiple datasets is crucial.

**Methods:**

We accessed and systematically investigated the content of 20 major AD cohort datasets at the data level. Both, a medical professional and a data specialist, manually curated and semantically harmonized the acquired datasets. Finally, we developed a platform that displays vital information about the available datasets.

**Results:**

Here, we present ADataViewer, an interactive platform that facilitates the exploration of 20 cohort datasets with respect to longitudinal follow-up, demographics, ethnoracial diversity, measured modalities, and statistical properties of individual variables. It allows researchers to quickly identify AD cohorts that meet user-specified requirements for discovery and validation studies regarding available variables, sample sizes, and longitudinal follow-up. Additionally, we publish the underlying variable mapping catalog that harmonizes 1196 unique variables across the 20 cohorts and paves the way for interoperable AD datasets.

**Conclusions:**

In conclusion, ADataViewer facilitates fast, robust data-driven research by transparently displaying cohort dataset content and supporting researchers in selecting datasets that are suited for their envisioned study. The platform is available at https://adata.scai.fraunhofer.de/.

**Supplementary Information:**

The online version contains supplementary material available at 10.1186/s13195-022-01009-4.

## Background

Alzheimer’s disease (AD) and dementia research has progressed considerably thanks to the increased availability of patient-level cohort datasets [1]. Cohort data have, among others, laid the foundation to discover novel biomarkers [2], investigate disease progression [3], and identify disease subtypes [4]. To ensure the robustness and reproducibility of results achieved in such data-driven analyses, they must be externally validated in independent cohort datasets [5]. Working across multiple cohort datasets is, however, impeded by several profound challenges. The first challenge manifests in the access to further validation cohort datasets, as third-party researchers have to go through time-intensive application processes that often span several weeks before they can actually start getting familiar with the acquired data. Secondly, once access is granted, the validation datasets have to be comparable to the original discovery dataset concerning their assessed variables [6]. This means that (1) a largely overlapping set of variables should have been measured in both cohorts and (2) these variables need to be harmonized across the independent cohort datasets, which is rarely the case by default. Identifying and semantically harmonizing equivalent variables in distinct datasets is an arduous task given that datasets typically employ their own variable naming system [7]. While theoretical guidelines for AD data harmonization have been previously proposed [8], as of now and to the best of our knowledge, no comprehensive mapping catalog is available to the AD research community that would help to unify the variable names across existing cohorts.

Across-cohort interoperability, however, goes beyond the semantic layer as statistical distributions of equivalent variables might differ among cohorts [9]. Our recent study revealed that such systematic statistical differences can bias results of data-driven analyses based on cohort data [10]. However, in practice, researchers only see the factual content of a shared dataset after data download occurred and data investigation started. At this stage, the realization of, for example, incompatible discovery and validation datasets can render the process of data access and exploration a waste of time as the lacking data interoperability would render the envisioned analysis infeasible.

Several funding bodies, for example, the Innovative Medicine Initiative (IMI) or the Alzheimer’s Disease Data Initiative (ADDI), have launched large projects to address data problems in the AD domain, for example, the European Medical Information Framework (EMIF) [11], ROADMAP [12], or the ADDI Workbench, and new calls were issued in this direction. In fact, both EMIF and ROADMAP have built information sources on cohort datasets that were assembled from the respective cohorts’ self-reported metadata [13, 14]. However, in a recent study, we observed that the information gained through such metadata-driven cohort assessments differs from the content that is factually shared with researchers after successful access applications [15].

In this work, we present ADataViewer, an interactive tool that enables the scientific community to explore 20 AD cohort datasets, both from a semantic and statistical perspective. To establish semantic interoperability across these datasets, we created a variable mapping catalog that harmonizes 1196 unique variables encountered in the datasets, spanning nine data modalities. Leveraging these semantically harmonized versions of the datasets, we developed tools and interfaces that facilitate the exploration of the cohort datasets with respect to longitudinal follow-up, demographics, ethnoracial diversity, measured modalities, and individual variables. Finally, we present ADataViewers’ “StudyPicker,” a tool that assists researchers in identifying cohort datasets suited for their envisioned analysis.

## Methods

### Harmonizing variables across cohorts

Semantic harmonization of the datasets was achieved through meticulous manual curation. Two curators systematically investigated variable names, metadata describing the variable content, and the values stored in the respective data tables across each dataset to gain robust mappings between equivalent variables. We opted for a multidisciplinary curation team to combine the complementary strengths of a curator from a medical background with those of a second curator leveraging a data-driven perspective. In the first step, the curators categorized the variables of each dataset according to a set of modalities (e.g., magnetic resonance imaging (MRI), demographics, and genotyping). To facilitate the curation process, mappings were proposed to the curators based on variable name similarity in modalities where the number of features was abundant. For the majority of modalities, we mapped approximately between 10 to 30 variables, with the exception being the MRI modality which comprised more than 1000 variables, as it contained a vast selection of brain region-specific measures derived from the raw images (e.g., volumes or thickness). No specific data model (e.g., FHIR or OMOP) was used. For more detailed curation guidelines, we refer to the Supplementary Material. Whenever possible, variables found in the investigated AD datasets were additionally mapped to ontologies that provided respective semantic context. Further details on the used ontologies and the process of mapping variable names to ontologies are described in the Supplementary Material.

### Data access and data privacy

ADataViewer does not store or enable the download of any cohort data itself. All displayed plots and provided exploration tools are fully anonymized and no participant identifying information is disclosed nor stored in the underlying database, not even the original study internal patient identifiers. Shown statistical plots are solely based on summary statistics or univariate analyses that cannot be linked to other variables or personal information. To facilitate access to the datasets, we provide links that lead researchers to the original data portals through which the respective cohorts are distributed.

## Results

ADataViewer is an interactive platform that enables the detailed exploration of, at the time of publication, 20 major cohort datasets from the AD domain. Its goal is to provide an overview across their content from a predominantly data-driven perspective. Each section of ADataViewer focuses on distinct aspects of the investigated datasets. The “Modality” section provides an overview of the data modalities collected in each cohort (e.g., magnetic resonance imaging (MRI), autopsy, and genotype data). The “Ethnicity” page displays the ethnoracial diversity in each cohort study as well as aggregated plots over specific geographic regions. In the “Longitudinal” section, the frequency and abundance of follow-up assessments are presented both per cohort and variable. The “Biomarkers” section allows the visualization of variable distributions and their comparison across cohorts. The semantic mappings between cohort name spaces are covered in the “Mappings” section. Finally, the “StudyPicker” leverages on all of these sections to guide researchers to the cohort datasets which provide the best basis for their planned analyses.

Instead of relying solely on study protocols and reported metadata, we based all our investigations on the data that were factually shared by the respective data owners. To transparently mirror the state of the dataset to which researchers will gain access after successful application, we refrained from any extensive data processing (e.g., transforming numerical ranges and value representations). As such, any inconsistencies in the datasets (e.g., extreme outliers) will be accordingly displayed in ADataViewers’ tools and visualizations. Consequently, this allows researchers to comprehensively evaluate the data that will actually be available for analysis.

### Accessed AD cohort datasets

To enable a comprehensive exploration of the available AD data, it was vital to identify, access, and curate as many cohort-level datasets as possible. Therefore, we systematically scanned data repositories and scientific publications, leading to the identification of 24 cohorts of which most claimed to follow the open science paradigm and share their data with third-party researchers. After applying for access to the corresponding data owners, we acquired 20 of those datasets over the course of 3 years (information on why the four remaining datasets were not accessed is provided in the Supplementary Material). These datasets originated from a heterogeneous pool of studies that followed a variety of different goals ranging from purely observational cohort studies over memory clinic data collections to dedicated clinical trials. Concordantly, the employed participant recruitment procedures, inclusion and exclusion criteria, and measured data modalities varied among them. More information about the collected datasets, their content, and original study aim is given in Table 1; for further study-specific details, we refer to the original publications.

**Table 1:** AD cohorts available for exploration using ADataViewer

Cohort	Consortium	Patients at baseline	Modalities	Longitudinal (yes/no)	Study type
A4 [16]	Anti-Amyloid Treatment in Asymptomatic Alzheimer’s Disease	6945	7	No^a^	Clinical trial
ABVIB [17]	Aging Brain: Vasculature, Ischemia, and Behavior	280	2	Yes	Observational study
ADNI [18]	The Alzheimer’s Disease Neuroimaging Initiative	2249	12	Yes	Observational study
AIBL [19]	The Australian Imaging, Biomarker & Lifestyle Flagship Study of Ageing	1378	9	Yes	Observational study
ANMerge [20]	AddNeuroMed	1703	10	Yes	Observational study
ARWIBO [21]	Alzheimer’s Disease Repository Without Borders	2617	10	Yes	Observational study
DOD-ADNI [22]	Effects of TBI & PTSD on Alzheimer’s Disease in Vietnam Vets	458	11	Yes	Observational study
EDSD [23]	The European DTI Study on Dementia	474	7	No	Observational study
EMIF-1000 [24]	European Medical Information Framework	1199	10	No	Meta-cohort
EPAD V.IMI [25]	European Prevention of Alzheimer’s Dementia	2096	9	Yes	Observational study
I-ADNI [26]	The Italian Alzheimer’s Disease Neuroimaging Initiative	262	5	No	Observational study
JADNI [27]	Japanese Alzheimer’s Disease Neuroimaging Initiative	567	9	Yes	Observational study
NACC [28]	The National Alzheimer’s Coordinating Center	40,948	11	Yes	Memory clinic database
OASIS-1 [29] and OASIS-2 [30]	Open Access Series of Imaging Studies	564	3	Yes	Observational study
PREVENT-AD [31]	Pre-symptomatic Evaluation of Experimental or Novel Treatments for Alzheimer’s Disease	348	8	Yes	Clinical trial
PharmaCog [32]	Prediction of Cognitive Properties of New Drug Candidates for Neurodegenerative Diseases in Early Clinical Development	147	6	Yes	Observational study
ROSMAP [33]	The Religious Orders Study and Memory and Aging Project	3626	7	Yes	Observational study
VASCULAR [34]	The Vascular Contributors to Prodromal Alzheimer’s disease	250	8	No	Non-interventional cohort study
VITA [35]	Vienna Transdanube Aging	606	5	Yes	Observational study
WMH-AD [36]	White Matter Hyperintensities in Alzheimer’s Disease	90	5	No	Observational study

A complete overview about the collected data modalities can be found under https://adata.scai.fraunhofer.de/modality

^a^Follow-up assessments were planned for A4 but no according data was released at the time of this publication

### Semantic harmonization of the accessed cohort datasets

**Fig. 1:**
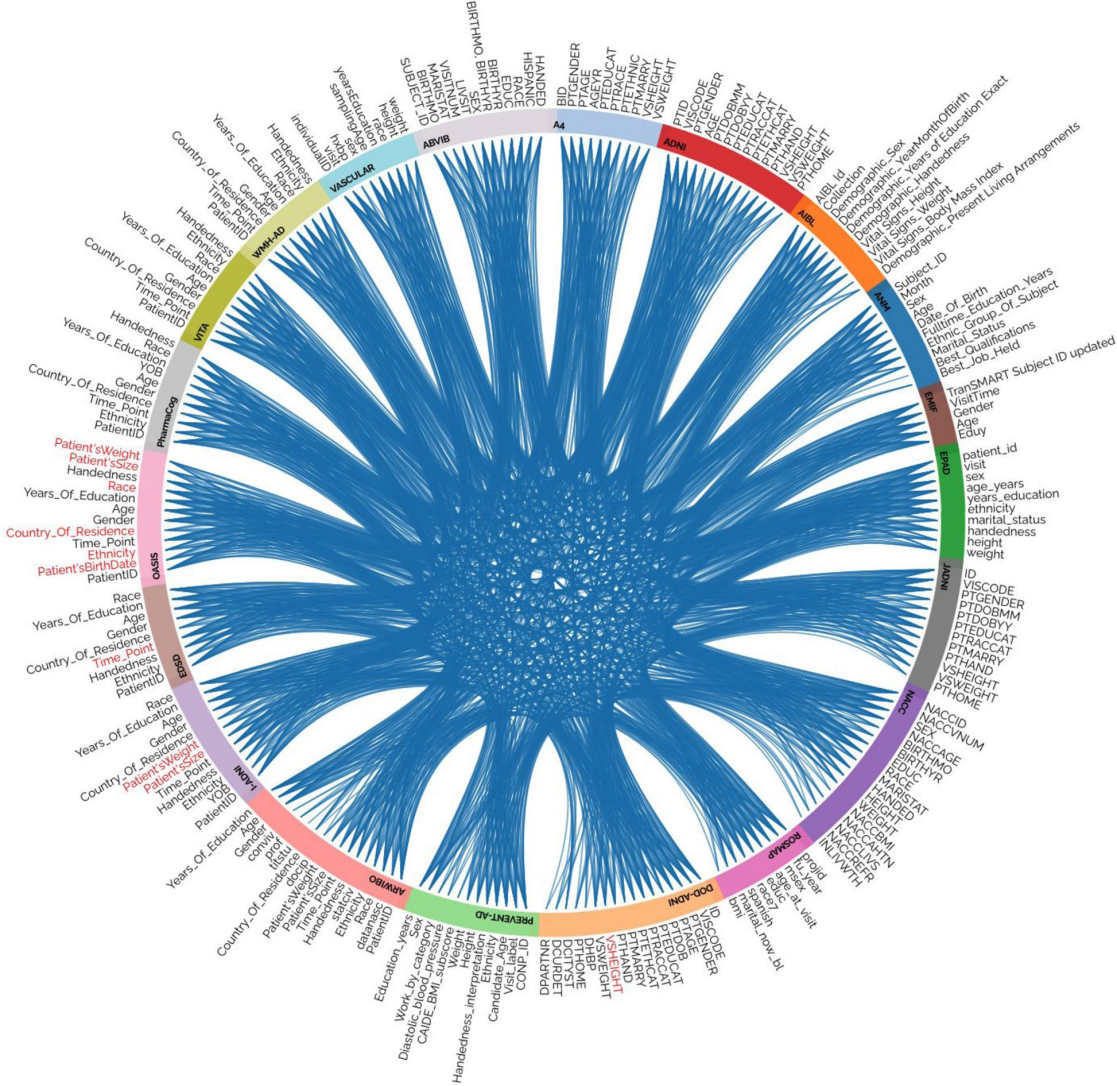
Mapping of demographic variables across the 20 cohorts. Red labels indicate variables mentioned in the metadata which consisted purely of missing data in the shared dataset. The corresponding plot for each modality as well as the underlying mapping tables for data harmonization are available at https://adata.scai.fraunhofer.de/mappings.

To build ADataViewer, we mapped 1196 unique terms across the investigated datasets corresponding to variables from nine different data modalities (Fig. 1). Table 2 shows the total number of mapped terms per modality and cohort. Furthermore, to connect the variables of the cohort datasets to clearly defined semantic concepts, we additionally mapped them to standardized ontologies. In total, 241 concepts from seven distinct referential ontologies were used in this process (more details in the Supplements). All mappings can be explored through interactive visualizations and tables at https://adata.scai.fraunhofer.de/mappings. The genotype and omics modalities of datasets were not mapped as they are already precisely defined by genetic database identifiers (e.g., rsID’s or UniProt identifiers) and their corresponding reference genome. A prerequisite for mapping the variables was that they were at least present in two independent cohorts.

**Table 2:** Number of mapped unique variables per cohort and modality

**Dataset**	**Demographics**	**Clinical**	**MRI**	**PET**	**CSF**	**Plasma**	**Comorbidities**	**Family**	**Lifestyle**
**A4**	13	5	44	1	0	0	2	6	4
**ABVIB**	12	0	0	0	0	0	0	0	0
**ADNI**	17	23	247	3	10	11	14	8	5
**AIBL**	15	16	3	2	3	0	12	2	5
**ANMerge**	14	11	136	0	0	0	1	3	1
**ARWIBO**	21	14	1026	21	3	6	13	3	2
**DOD-ADNI**	21	20	249	1	3	0	18	6	6
**EDSD**	12	8	1026	8	3	2	4	2	0
**EMIF-1000**	8	4	3	1	6	0	3	0	4
**EPAD V.IMI**	14	11	80	0	3	0	17	5	4
**I-ADNI**	15	10	1026	8	3	1	1	2	0
**JADNI**	15	21	871	2	3	0	14	6	4
**NACC**	20	17	123	2	3	0	14	3	6
**OASIS**	16	3	1026	8	3	2	0	2	0
**PREVENT-AD**	15	4	0	0	7	0	5	5	0
**PharmaCog**	13	16	1026	8	3	2	0	2	0
**ROSMAP**	12	9	0	0	0	0	8	0	1
**VASCULAR**	9	8	31	0	0	0	3	0	2
**VITA**	12	3	1026	8	3	2	0	2	0
**WMH-AD**	12	4	1025	8	3	2	0	2	0
**Total unique terms**	**23**	**34**	**1050**	**24**	**14**	**15**	**20**	**9**	**7**

### The StudyPicker: variable-based selection of cohort datasets

The StudyPicker is a tool that supports researchers in finding datasets based on the requirements of their envisioned analysis (https://adata.scai.fraunhofer.de/study_picker). It takes a collection of variable names as input and ranks the cohorts in ADataViewer based on the availability of these specified variables (Fig. 4A). The generated ranking shows the availability of the variables and the number of participants per cohort for whom these variables have been assessed at the study baseline, as well as their longitudinal coverage (i.e., assessment frequency and the number of participants assessed per visit) (Fig. 4B). Additionally, links are provided that guide interested researchers directly to the data access applications of the respective datasets. The StudyPicker is particularly helpful for hypothesis-driven research or validation studies in which the variables that are elementary to conduct the planned analysis are often known in advance.

### Detailed exploration of dataset content through interactive visualizations

Next to the semantic perspective, ADataViewer also allows for a detailed exploration of the integrated datasets based on descriptive statistics. Statistical distributions of numerical and categorical variables of interest can be visualized and compared across the available cohorts (https://adata.scai.fraunhofer.de/biomarkers). This functionality enables comparisons between individual diagnosis groups (i.e., cognitively unimpaired (CU), mild cognitive impairment (MCI), AD) as well as the complete cohorts. Using these visualizations, researchers can investigate distributions and value representations encountered in the datasets and identify possible differences among them before starting their analysis.

**Fig. 2:**
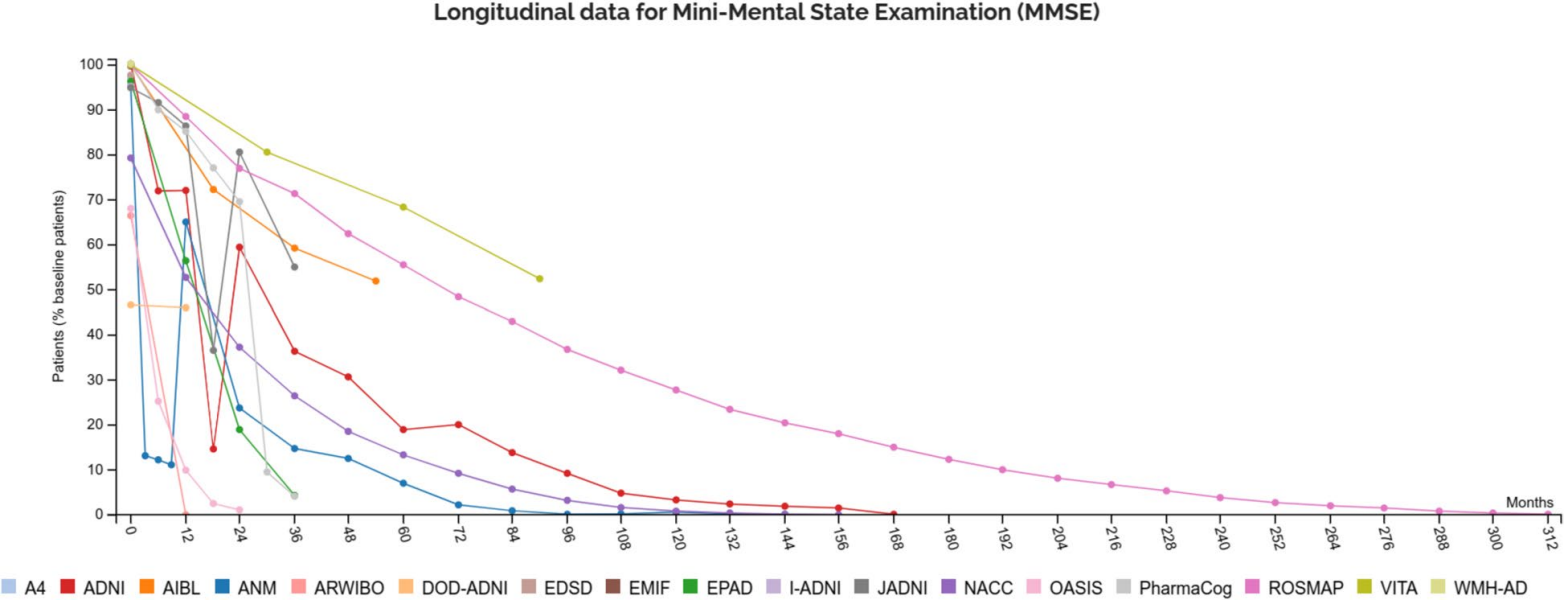
Exemplary longitudinal plot of MMSE assessments generated using ADataViewer. Displayed are cohorts and their respective number of assessed participants for the selected variable

A longitudinal view of the data can be generated in the “Longitudinal” section. Dedicated visualizations display the follow-up per cohort on a variable level (Fig. 2).

### Meta-analysis of cohort study content, assessed variables, and common modalities

**Fig. 3:**
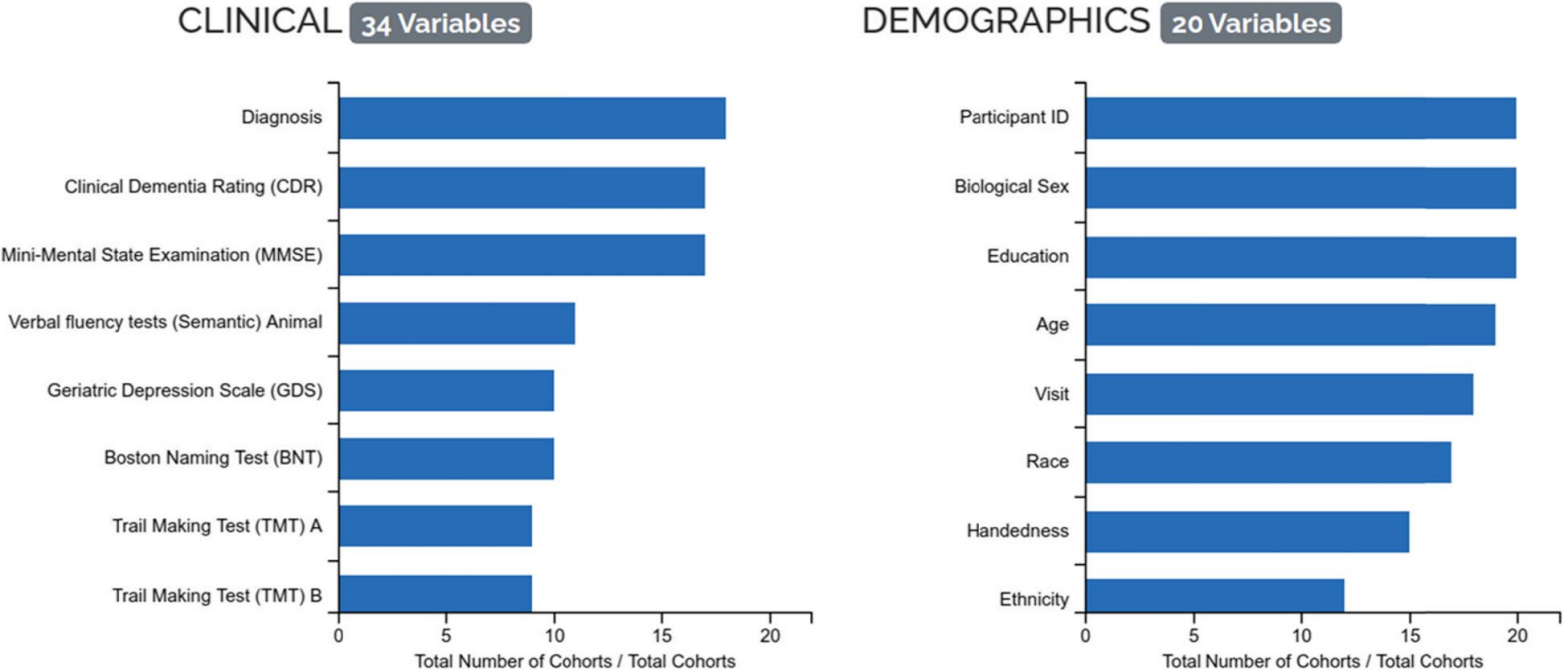
Assessment frequency of exemplary variables across cohorts. Interactive figure displaying the number of studies in which each specific variable was encountered (https://adata.scai.fraunhofer.de/biomarkers)

Besides the exploration and comparison of specific cohorts, ADataViewer helps to get a comprehensive overview of the state of the data landscape formed by the underlying cohorts. Here, the modality map (https://adata.scai.fraunhofer.de/modality) displays how commonly specific data modalities were included in cohort studies and, simultaneously, highlights areas that currently remain underexplored. Along the same line, Fig. 3 shows an excerpt from an interactive visualization that depicts how many studies measured each individual variable. Furthermore, the plots displaying the ethnoracial diversity encountered in each individual cohort, and across cohorts grouped by geographic location, reveal over- and underrepresentation of ethnoracial groups in data-driven AD research. All of this information can be vital when designing a novel cohort study aiming either for compatibility to other studies or at illuminating blind spots previously underrepresented in the AD data landscape.

### Exemplary application scenarios employing ADataViewer

While there are multiple scenarios in which ADataViewer can support AD research, we focus on two scenarios below. Another application scenario not explained here, however, one that would follow similar routes as the ones outlined below, would be the writing of grant applications and identifying datasets to include into the proposal.

#### Scenario 1

A researcher is searching for a discovery and validation cohort to model cognitive decline in the light of hippocampus atrophy, amyloid PET, and depression. The variables of interest are the Mini-Mental State Examination (MMSE), Clinical Dementia Rating Sum of Boxes (CDRSB), hippocampus volume, Amyvid Positron Emission Tomography (AV PET), Geriatric Depression Scale (GDS), and variables to correct for possible confounding (age, biological sex, education, and APOE ε4 allele presence).

**Fig. 4:**
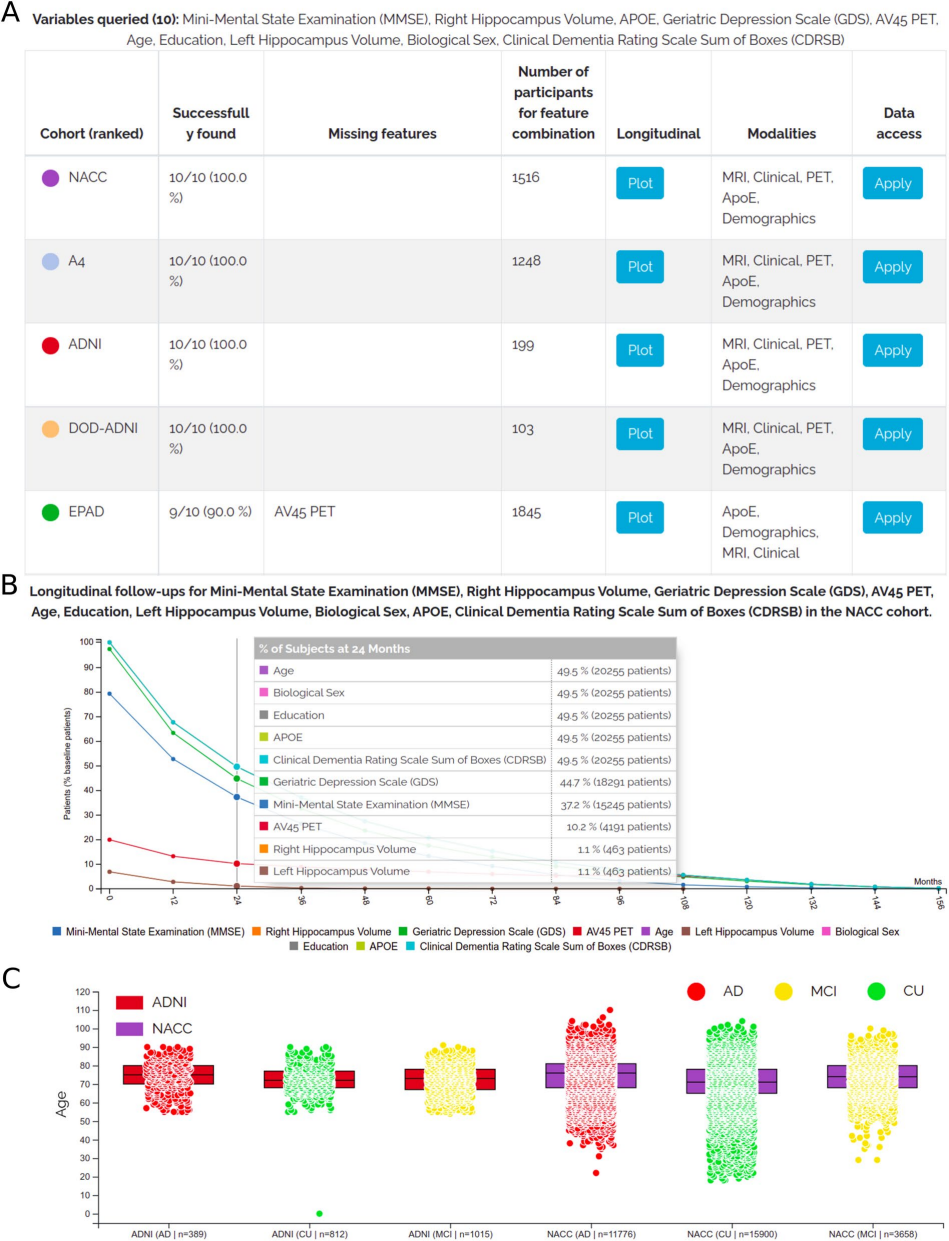
Using ADataViewer to identify suitable cohort datasets in a use case scenario. Selection of this case scenario was with the aim to evaluate cognitive decline in the light of depression, AV PET, and hippocampal atrophy. All graphs were created using the tools of ADataViewer. **A** Excerpt of the ranking received by entering the variables of interest specified in application scenario 1 into the StudyPicker. **B** Longitudinal coverage of the specified variables in the NACC cohort. See Fig. S1 for the other cohorts’ plots. **C** Comparison of the age distributions encountered across diagnostic groups of ADNI and NACC

Given such a set of variables of interest, the StudyPicker of ADataViewer is the appropriate starting point to identify relevant cohorts. After submitting the variable query, we can directly observe that NACC, A4, ADNI, and DOD-ADNI contain all specified variables of interest (Fig. 4A). However, after inspecting the follow-up plots, it is revealed that only NACC and ADNI hold sufficient longitudinal data to detect time-dependent relationships (here, 463 and 557 patients over 24 months of study runtime, respectively) (Fig. 4B and Fig. S1). Besides these two cohorts, EPAD, including 1845 participants, could also provide a rich basis for the planned analysis if AV PET would be omitted (Fig. 4A).

For a final evaluation on whether NACC and ADNI would suit the study needs, the “Biomarkers” section can be used to compare cohort demographics and variable distributions. For example, comparing the age of participants in NACC and ADNI reveals a higher variance in the NACC data and the presence of younger participants who would have been excluded from the ADNI study (Fig. 4C). Furthermore, investigating the hippocampal volumes exposes a difference in value representation between the cohorts, as NACC values have been reported as normalized values (Fig. S2). Consequently, it could be concluded that both datasets could be viable options for the discovery and replication process of a data-driven study, given that the representations of the hippocampal volume can be unified. Finally, the application process for data access can be initiated directly through the StudyPicker.

#### Scenario 2

A consortium is planning to conduct a longitudinal cohort study that aims at investigating AD in previously underrepresented ethnoracial groups. The assessed variables, however, should be compatible with other landmark AD cohorts to allow for a comparison of achieved results.

First, the ethnoracial diversity encountered across previous AD cohorts can be explored in the “Ethnicity” section of ADataViewer. Their investigation demonstrates that 19 of the 20 cohorts enrolled predominantly caucasian/white participants. Keeping our proposed study goals in mind, it would therefore make sense to exclude caucasian/white participants from the recruitment of the envisioned study to focus on the currently underrepresented groups.

To achieve high compatibility with previous AD studies, the planned study should align its follow-up intervals and the assessed variables/data modalities to them. Here, the data modality map indicates that we should include demographics, clinical assessments, MRI, cerebrospinal fluid (CSF) biomarkers, at least APOE genotyping, administered medication, comorbidities, and the family history of participants to achieve a strong overlap in data modalities (Fig. S3). More specifically, the most prominently assessed variables per modality can be explored in the “Biomarkers” section (Fig. 3). For example, we can observe that Clinical Dementia Rating (CDR) and MMSE are the most conducted cognitive assessments; demographics most commonly cover the biological sex, age, years of education, and ethnoracial group of participants; and phosphorylated tau, total tau, and beta-amyloid were abundantly measured as CSF markers. By leveraging this information, we can make an informed decision on the variables we want to measure in the envisioned cohort study, such that an exploration of AD progression is feasible and that possible differences to cohorts of other ethnoracial compositions can be systematically evaluated. Additionally, the value ranges commonly encountered per variable can be explored using the biomarker boxplots (Fig. 4C). Once the cohort study was conducted, we can use the provided variable mapping catalog to harmonize the new cohort dataset to all 20 datasets currently present in ADataViewer.

## Discussion

ADataViewer aims at advancing patient data-driven AD research by increasing the findability and interoperability of cohort datasets and providing a deeper understanding of their content, both from a semantic and statistical perspective. The platform supports the variable-level exploration of 20 AD cohort datasets and enables researchers to identify datasets suited for their envisioned studies before spending time on data access applications. In this context, we created, to the best of our knowledge, the most comprehensive variable mapping catalog in the AD domain that semantically harmonizes 1196 unique variables across all investigated cohorts.

Aspiring to contribute to a FAIR data paradigm (findable, accessible, interoperable, reusable) in AD research [37], ADataViewer increases the findability of AD cohort datasets by displaying and suggesting possible data resources to researchers, enables better accessibility through direct links to the respective data access points, provides the variable mapping catalog to establish data interoperability, and facilitates the reuse of data for validation purposes. We believe that the presented platform can elevate data-driven AD research to be faster and more robust, because it becomes significantly easier to access the right datasets and validate results across multiple independent cohorts. In turn, this will help to better understand the heterogeneity across AD patients [38] and help to reveal possible cohort-specific findings [10].

Collecting patient-level data is a vastly expensive process. Therefore, studies are often limited concerning their sample size, follow-up time, and variety of assessed data modalities. ADataViewer transparently provides researchers with information about what they can expect from specific datasets and whether it makes sense for them to spend a substantial amount of time on the acquisition of the individual data resource. Limiting the time spent on unfruitful dataset acquisitions will accelerate and benefit the actual analysis of the data. On this note, we would like to emphasize that ADataViewer is not meant to promote only the largest, most complete cohorts, but to show all available datasets that contain the information of interest for a conceived project. While larger cohorts often fare better as discovery cohorts, any cohort with equivalent information, regardless of the size, could present a valuable resource for the subsequent validation of results and should therefore be considered.

Given the restrictions of sensible personal data, there are multiple initiatives testing and establishing federated learning concepts that aim to facilitate secure remote access to multiple sensible datasets [39]. These concepts rely on interoperable data and our mappings and data descriptions could provide a starting point to establish such comprehensive interoperability by extending them into a complete data model following, for example, the OMOP or FHIR standard.

We plan to update ADataViewer as well as its underlying information (e.g., the mappings) whenever we get access to new datasets. However, an automatic periodic updating is infeasible, as the data is usually not shared via programmatic interfaces but through personal contacts and access-restricted data portals.

### Limitations

One strength and simultaneous limitation of this work was its overarching premise that the data investigation was not based purely on descriptive metadata but on the dataset that was factually shared with us. Therefore, all results are based on the status of the distributed data and could vary from the content mentioned in official study reports or other versions of the same dataset. Ultimately, however, what drives the advancement of AD research is the factually shared, analyzable data and not what could potentially be available in theory.

The decision on how strict equivalence of variables is defined inevitably remains arbitrary to some degree. Here, we define two variables as semantically equivalent if the same information is presented in principle (i.e., the content of both variables can at least be broken down into the same information, see Supplementary Material for examples). Therefore, the acquisition method (e.g., type of MRI scanner) between two variables that were declared to be semantically equivalent may still differ and subsequent pre-processing of the raw data might be necessary to account for resulting statistical differences (e.g., elimination of batch effects). Sharing statistically harmonized data via ADataViewer is infeasible due to legal data sharing restrictions. However, the presented semantic mapping catalog presents a starting point to directly identify equivalent variables of interest and initiate the following pre-processing steps.

## Conclusion

With ADataViewer, we aim to contribute to a robust, data-driven research culture that carefully reproduces and validates scientific results across multiple comparable datasets. As such, instead of pointing towards a single data resource, ADataViewer transparently displays the content of all integrated AD cohort datasets and the StudyPicker proposes all of these resources that match the researcher’s requirements. Our provided variable mappings build the basis for in-depth dataset comparisons and can act as a starting point to select and harmonize suited discovery and validation datasets.

## Supplementary Information

**Additional file 1: Figure S1.** Longitudinal follow-up plots the specified variables in a case scenario. **Figure S2.** Distribution of hippocampus volume displayed with boxplots using the “Biomarkers” tool of the ADataViewer. **Figure S3.** The modality map, describing which data modalities have been assessed per cohort.

## Data Availability

All investigated datasets used in this study can be obtained from the respective data owners. Links are provided at https://adata.scai.fraunhofer.de/cohorts.

## Abbreviations

A4: Anti-Amyloid Treatment in Asymptomatic Alzheimer’s Disease
ABVIB: Aging Brain Vasculature, Ischemia, and Behavior
AD: Alzheimer’s Disease
ADDI: Alzheimer’s Disease Data Initiative
ADNI: Alzheimer’s Disease Neuroimaging Initiative
AIBL: Australian Imaging, Biomarker & Lifestyle Flagship Study of Ageing
ANMerge: AddNeuroMed
ARWIBO: Alzheimer’s Disease Repository Without Borders
AV PET: Amyvid Positron Emission Tomography
CDR: Clinical Dementia Rating
CDRSB: Clinical Dementia Rating Sum of Boxes
CSF: Cerebrospinal Fluid
CU: Cognitively Unimpaired
DOD-ADNI: Effects of TBI & PTSD on Alzheimer’s Disease in Vietnam Vets
EDSD: European DTI Study on Dementia
EMIF: European Medical Information Framework
EPAD: European Prevention of Alzheimer’s Dementia
GDS: Geriatric Depression Scale
I-ADNI: Italian Alzheimer’s Disease Neuroimaging Initiative
IMI: Innovative Medicine Initiative
JADNI: Japanese Alzheimer’s Disease Neuroimaging Initiative
MCI: Mild Cognitive Impairment
MMSE: Mini-Mental State Examination
MRI: Magnetic Resonance Imaging
NACC: National Alzheimer’s Coordinating Center
OASIS: Open Access Series of Imaging Studies
PREVENT-AD: Pre-symptomatic Evaluation of Experimental or Novel Treatments for Alzheimer’s Disease
PharmaCog: Prediction of Cognitive Properties of New Drug Candidates for Neurodegenerative Diseases in Early Clinical Development
ROSMAP: Religious Orders Study and Memory and Aging Project
VASCULAR: Vascular Contributors to Prodromal Alzheimer’s Disease
VITA: Vienna Transdanube Aging
WMH-AD: White Matter Hyperintensities in Alzheimer’s Disease
