# Supplementary material for: ADataViewer: exploring semantically harmonized Alzheimer’s disease cohort datasets
Source: Alzheimers Res Ther. 2022 May 21;14:69. doi: 10.1186/s13195-022-01009-4 (PMC9123725; doi:10.1186/s13195-022-01009-4)
Supplement: Supplementary file 1 — Additional file 1: Figure S1. Longitudinal follow-up plots the specified variables in a case scenario. Figure S2. Distribution of hippocampus volume displayed with boxplots using the “Biomarkers” tool of the ADataViewer. Figure S3. The modality map, describing which data modalities have been assessed per cohort. [file 13195_2022_1009_MOESM1_ESM.docx]

**Supplementary Material**

**ADataViewer: Exploring Semantically Harmonized Alzheimer’s Disease Cohort Datasets**

**Yasamin Salimi^1,2^, Daniel Domingo-Fernández^1^, Carlos Bobis-Álvarez^3^, Martin Hofmann-Apitius^1,2^, Colin Birkenbihl^1,2^ for the Alzheimer's Disease Neuroimaging Initiative^^[[1]](#footnote-1)^^, the Japanese Alzheimer’s Disease Neuroimaging Initiative^^[[2]](#footnote-2)^^, for the Aging Brain: Vasculature, Ischemia, and Behavior Study^^[[3]](#footnote-3)^^, the Alzheimer's Disease Repository Without Borders Investigators^^[[4]](#footnote-4)^^, for the European Prevention of Alzheimer’s Disease (EPAD) Consortium^^[[5]](#footnote-5)^^**

1. Department of Bioinformatics, Fraunhofer Institute for Algorithms and Scientific Computing (SCAI), Sankt Augustin 53754, Germany
2. Bonn-Aachen International Center for IT, Rheinische Friedrich-Wilhelms-Universität Bonn, Bonn 53115, Germany
3. University Hospital Ntra. Sra. de Candelaria, Santa Cruz de Tenerife 38010, Spain.

Corresponding author: Yasamin Salimi (yasamin.salimi@scai.fraunhofer.de)

# Dataset access rejections

The four additional datasets we applied for and their responses to our data access requests are listed below:

- The Dominantly Inherited Alzheimer Network (DIAN): Rejected because “The request, in its current form, does not meet the scope set out by the DIAN study.” [1]
- MEMENTO: No reply as of now (one year after access application) [2]
- Genetic and Environmental Risk in Alzheimer’s Disease (GERAD): No reply as of now (one year after access application) [3]
- BioFINDER: Rejected because “The data is unfortunately not publicly available.” (www.biofinder.se)

# Variable namespace harmonization

While some variables could be mapped unambiguously between the datasets (e.g. the MMSE or other standardized clinical assessments), others led to a trade-off between semantic accuracy and a complete mapping. For example, given studies measured the smoking behavior of participants, we could observe that the corresponding information was reported in varying detail: study A would report a binary variable “smoking yes/no”, study B would provide ordinal information “participant smokes none/1-3 cigarretes/4-10 cigarretes/>10 cigarettes per day”, while study C might have posed the question whether participants “smoked at least one cigarette over the last week”. While these variables measured three different aspects of smoking in the strict sense, we still mapped them together as their information could be made equivalent through appropriate pre-processing (here, the largest common denominator between those variables would be a binary indication whether the participant smokes at least occasionally).

# Linking mapped variables to referential ontologies

During the mapping process, we also linked variables to referential ontologies with the goal of cross-reference these variables with existing controlled vocabularies. Thus, each of the final variables were queried against definitions present in any of the ontologies indexed by the Ontology Lookup Service (OLS) (<https://www.ebi.ac.uk/ols/index>). In case of multiple matches, we preferably linked the variables to referential ontologies as they are constantly updated and contain cross-references to similar vocabularies. For instance, MONDO, which was used for diseases, contains cross-references to multiple ontologies. Thus, by mapping to MONDO, we are simultaneously mapping to further ontologies such as MeSH, DOID, and OMIM. The same principle applies to the other used ontologies, such as NICT, EFO, HP, and UBERON. In total, we linked the variables in ADataViewer to 7 distinct ontologies: Uber-anatomy Ontology (UBERON), Foundational Model of Anatomy Ontology (FMA), Ontology for MIRNA Target (OMIT), National Cancer Institute Thesaurus (NCIT), Experimental Factor Ontology (EFO), Human Phenotype (HP), and Mondo Disease Ontology (MONDO).

**Code**

All the essential scripts for preparation of the investigated datasets and extracting the results can be viewed:

Salimi, Y. (2021, October 14). ADataViewer: Exploring the Alzheimer's Disease Data Landscape (Version 1.0.0) [Computer software]. Zenodo. <https://doi.org/10.5281/zenodo.5570835>

**Figure S1:** Longitudinal follow-up plots the specified variables in a case scenario.


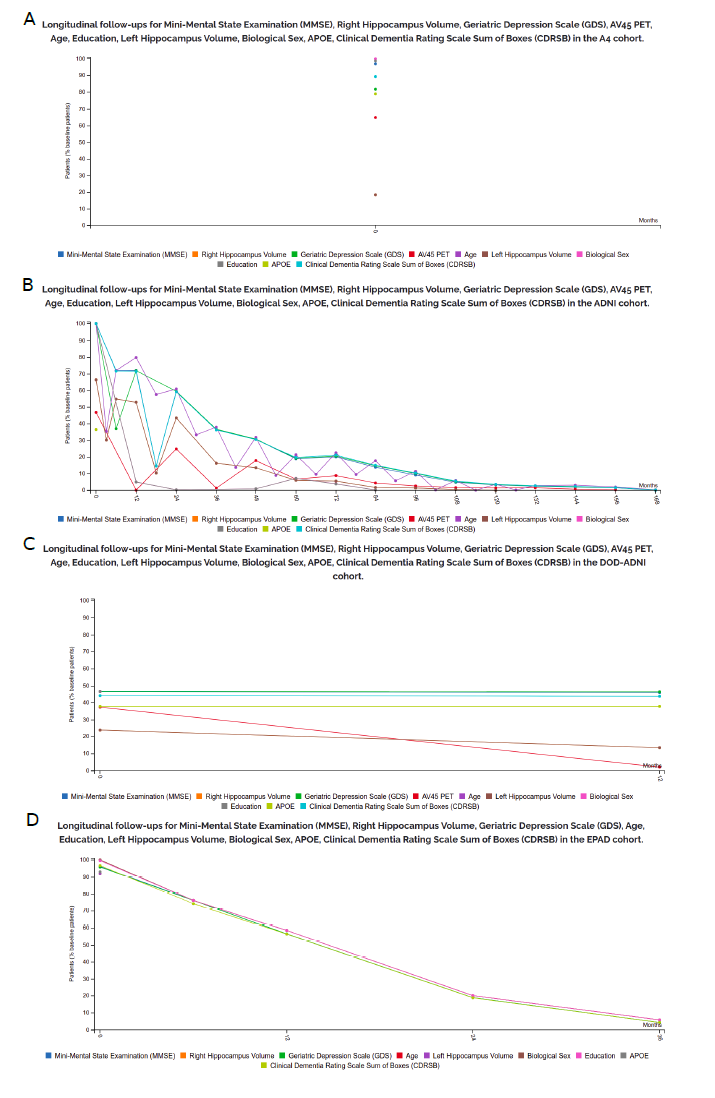


Plots corresponding to the StupyPicker application scenario for A, the NACC cohort. B, the A4 cohort. C, the ADNI cohort. D, the DOD-ADNI cohort.

**Figure S2:** Distribution of hippocampus volume displayed with boxplots using the "Biomarkers" tool of the ADataViewer.


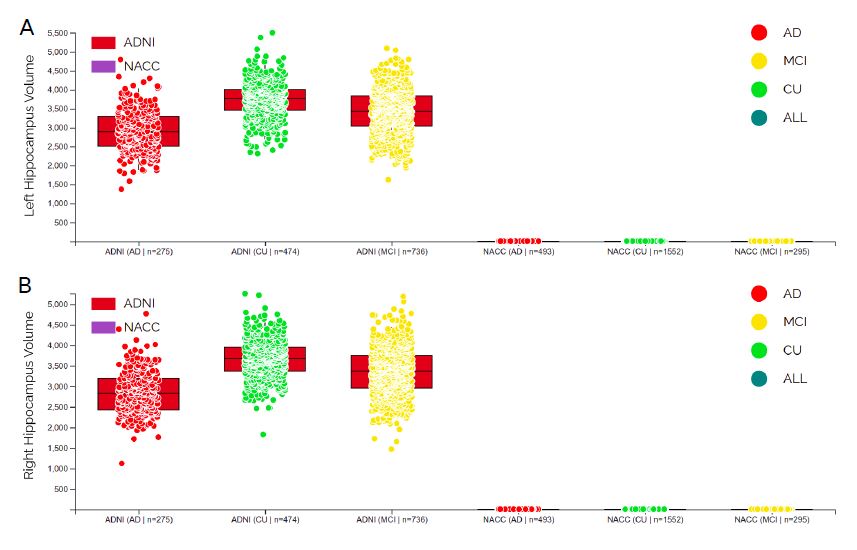


A, Left hippocampus volume across diagnostic groups of ADNI and NACC. B, Right hippocampus volume across diagnostic groups of ADNI and NACC.

**Figure S3:** The modality map, describing which data modalities have been assessed per cohort.

**
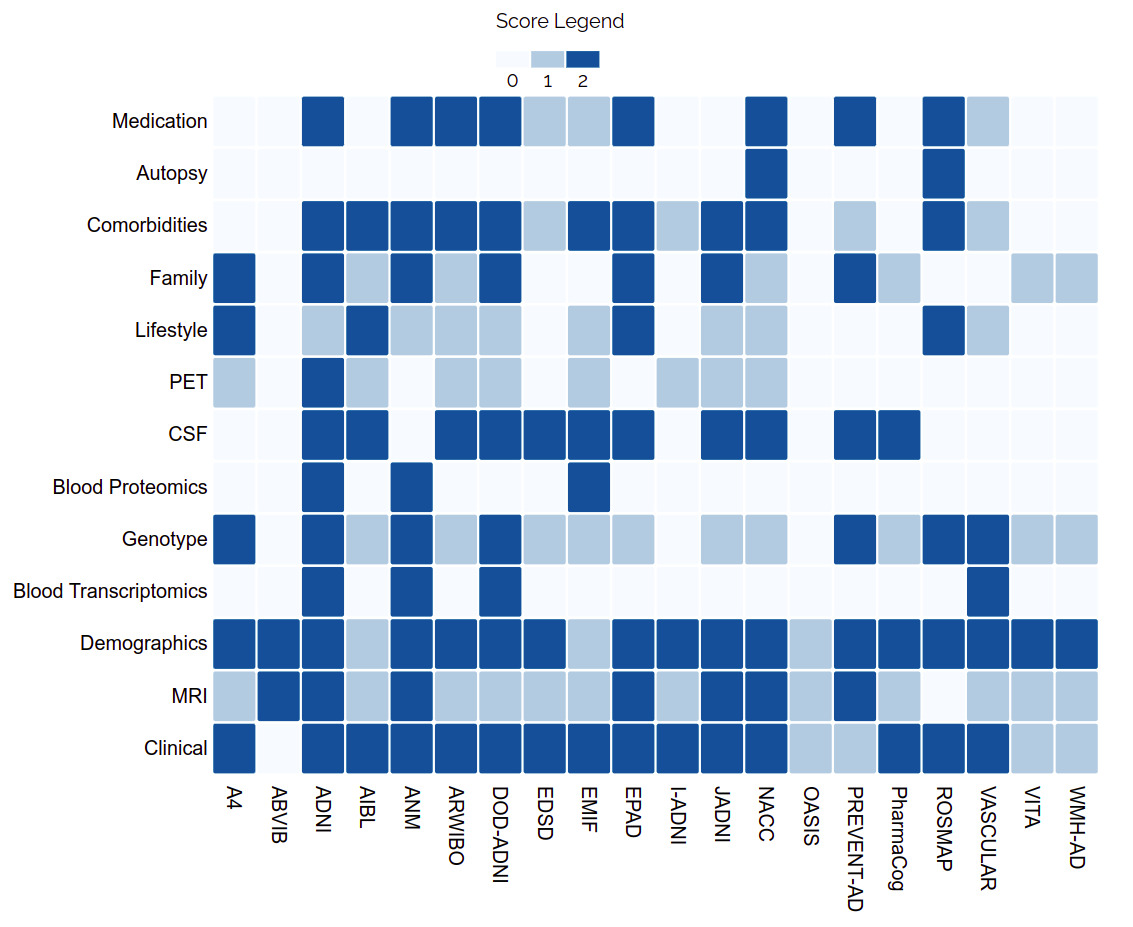
**

A detailed explanation of the scores is provided under [https://adata.scai.fraunhofer.de/modality#criteria](https://adata-dev.scai.fraunhofer.de/modality#criteria). In brief, a score of 0 means the modality was not measured, 1 means was partially measured and/or shared, 2 means it was available.

**References:**

1. Morris JC, Aisen PS, Bateman RJ, Benzinger TL, Cairns NJ, Fagan AM, et al. Developing an international network for Alzheimer research: The Dominantly Inherited Alzheimer Network. Clin Investig (Lond). 2012;2(10):975-984.
2. Dufouil C, Dubois B, Vellas B, Pasquier F, Blanc F, Hugon J, et al. Cognitive and imaging markers in non-demented subjects attending a memory clinic: study design and baseline findings of the MEMENTO cohort. Alzheimers Res Ther. 2017;9(1):67.
3. Hollingworth P, Sweet R, Sims R, Harold D, Russo G, Abraham R, et al. Genome-wide association study of Alzheimer's disease with psychotic symptoms. Mol Psychiatry. 2012;17(12):1316-1327.

1. Alzheimer’s Disease Neuroimaging Initiative: Data used in the preparation of this article were obtained from the Alzheimer’s Disease Neuroimaging Initiative (ADNI) database (adni.loni.usc.edu). As such, the investigators within the ADNI contributed to the design and implementation of ADNI and/or provided data but did not participate in analysis or writing of this report. A complete listing of ADNI investigators can be found at http://adni.loni. usc.edu/wp-content/uploads/how_to_apply/ ADNI_Acknowledgement_List.pdf [↑](#footnote-ref-1)
2. Japanese Alzheimer’s Disease Neuroimaging Initiative: Data used in preparation of this article were obtained from the Japanese Alzheimer’s Disease Neuroimaging Initiative (J-ADNI) database deposited in the National Bioscience Database Center Human Database, Japan (Research ID: hum0043.v1, 2016). As such, the investigators within J-ADNI contributed to the design and implementation of J-ADNI and/or provided data but did not participate in analysis or writing of this report. A complete listing of J-ADNI investigators can be found at: https://humandbs.biosciencedbc.jp/ en/hum0043-j-adni-authors. [↑](#footnote-ref-2)
3. Data used in preparation of this article were obtained from the Aging Brain: Vasculature, Ischemia, and Behavior Study (ABVIB). As such, the key investigators within the ABVIB contributed to the design and implementation of ABVIB and/or provided data but did not participate in analysis or writing of this report: Helena C. Chui M.D. (Principal Investigator), Charles C. DeCarli, M.D., William G. Ellis, M.D., William J. Jagust, M.D., Joel H. Kramer, Ph.D., Meng Law, M.D., Dan Mungas Ph,D. Bruce R. Reed, Ph.D., Nerses Sanossian, M.D., Michael W. Weiner, M.D. Wendy J. Mack, Ph.D., Harry V. Vinters, M.D., Chris Zarow, Ph.D., Ling Zheng, Ph.D. [↑](#footnote-ref-3)
4. Data used in preparation of this article were obtained from the Alzheimer's Disease Repository Without Borders (ARWiBo) database (www.arwibo.it). As such, the researchers within the ARWiBo contributed to the design and implementation of ARWiBo and/or provided data but did not participate in analysis or writing of this report. A complete listing of ARWiBo researchers can be found at: www.arwibo.it/acknowledgement.it [↑](#footnote-ref-4)
5. Data used in preparation of this article were obtained from the Longitudinal Cohort Study (LCS), delivered by the European Prevention of Alzheimer’s Disease (EPAD) Consortium. As such investigators within the EPAD LCS and EPAD Consortium contributed to the design and implementation of EPAD and/or provided data but did not participate in analysis or writing of this report. A complete list of EPAD Investigators can be found at: http://ep-ad.org/wp-content/uploads/2020/12/202010_List-of-epadistas.pdf [↑](#footnote-ref-5)
